# Liver fibrosis regression and progression during controlled hepatitis B virus infection among HIV–HBV patients treated with tenofovir disoproxil fumarate in France: a prospective cohort study

**DOI:** 10.7448/IAS.20.1.21426

**Published:** 2017-02-28

**Authors:** Anders Boyd, Julie Bottero, Patrick Miailhes, Caroline Lascoux-Combe, Hayette Rougier, Pierre-Marie Girard, Lawrence Serfaty, Karine Lacombe

**Affiliations:** ^a^ INSERM, UMR_S1136, Institut Pierre Louis d’Epidémiologie et de Santé Publique, Paris, France; ^b^ Service des Maladies Infectieuses et Tropicales, Hôpital Saint-Antoine, AP-HP, Paris, France; ^c^ Service des Maladies Infectieuses et Tropicales, Hôpital de la Croix-Rousse, Hospices Civils de Lyon, Lyon, France; ^d^ Service des Maladies Infectieuses et Tropicales, Hôpital Saint-Louis, AP-HP, Paris, France; ^e^ Institut de Médecine et d’Epidémiologie Appliquée, Paris, France; ^f^ Sorbonne Universités, UPMC Univ Paris 06, UMR_S 1136, Institut Pierre Louis d’Epidémiologie et de Santé Publique, Paris, France; ^g^ Service Hépato-gastro-entérologie, Hôpital Saint-Antoine, Paris, France

**Keywords:** noninvasive markers, liver fibrosis, liver cirrhosis, hepatocellular carcinoma, immunosuppression

## Abstract

**Introduction**: Long-term tenofovir disoproxil fumarate (TDF) use has been associated with significant regression of liver fibrosis during hepatitis B virus (HBV) mono-infection, yet little is known during HIV–HBV coinfection. The aim of this study was to evaluate the evolution of liver fibrosis and its determinants in TDF-treated coinfected patients.

**Methods**: In this prospective cohort study, 167 HIV–HBV-infected patients initiating TDF-containing antiretroviral therapy were included. Fibrosis was assessed using the FibroTest® at baseline and every six to twelve months. Risk factors for fibrosis progression (F0–F1–F2 to F3–F4) and regression (F3–F4 to F0–F1–F2) were evaluated.

**Results**: At baseline, 134 (80.2%) patients had detectable HBV-DNA (median = 4.93 log_10_ IU/mL, IQR = 2.94–7.15) and 104 (62.3%) had hepatitis B “e” antigen-positive serology. Median follow-up was sixty months (IQR = 36–93). In the 47 (28.1%) patients with F3–F4 baseline fibrosis, 7/47 (14.9%) regressed to F0–F1–F2 at last follow-up visit. Fibrosis regression was significantly associated with higher CD4+ cell counts (*P *= 0.009) and lower fasting triglyceride levels (*P *= 0.007) at TDF-initiation. In the 120 (71.9%) patients with F0–F1–F2-baseline fibrosis, 20/120 (16.7%) progressed to F3–F4 at last follow-up visit. Fibrosis progression was associated with male gender (*P *= 0.01), older age (*P *= 0.001), from low/moderate HBV-endemic country (*P *= 0.007), lower nadir CD4+ cell count (*P *= 0.03), higher fasting glycaemia (*P *= 0.03) and anaemia (*P *= 0.004) at TDF-initiation. Control of HBV replication at end of follow-up was extensive (88.1%), while no HBV-related factors emerged as predictors of progression/regression. Incidence of severe liver-related events was low (*n *= 4, rate = 0.5/100 person-years).

**Conclusions**: Liver fibrosis levels are stable for most coinfected patients undergoing TDF, despite control of HBV replication. Nevertheless, a concerning amount of liver fibrosis progression did occur, which could be partly explained by metabolic abnormalities and past severe immunosuppression and requires further evaluation.

## Introduction

Active replication of hepatitis B virus (HBV) is associated with several important causes of liver-related morbidity and mortality, strongly contending the need for its suppression [1]. Tenofovir disoproxil fumarate (TDF) has been established as an effective agent against long-term viral replication, while the risk of developing HBV resistance mutations is virtually null [2,3]. Consequently, TDF therapy in HBV mono-infected patients has been strongly associated with declines of biopsy-diagnosed liver fibrosis [4]. Preliminary evidence also suggests a substantial reduction, yet not complete elimination, in the risk of hepatocellular carcinoma (HCC) among TDF-treated HBV mono-infected patients [5].

For individuals coinfected with HIV and HBV, TDF represents an ideal component of antiretroviral therapy (ART) due to its potent efficacy against both HIV and HBV replication [6,7]. Studies among coinfected patients have indeed suggested a short-term clinical benefit of TDF-containing ART in liver fibrosis regression [8–10], yet these data do not extend past three years of treatment. This is particularly concerning for coinfected patients as HIV-associated immunosuppression could affect liver repair in the long term [11]. In addition, the lack of follow-up and relatively small changes of fibrosis measures in these studies have made it difficult to establish more clinically meaningful determinants associated with liver fibrosis and the possible impact this may have on liver-related morbidity and mortality.

Our study group has previously evaluated the effect of TDF on liver fibrosis within the coinfected population, yet this study, along with the other limitations mentioned above, included patients with hepatitis C virus (HCV) and/or hepatitis D virus (HDV) infection [8]. By prolonging follow-up almost twofold, increasing patient size and not including HCV/HDV-infected individuals, we aimed to more thoroughly describe the long-term evolution of liver fibrosis, using a noninvasive marker, in HIV–HBV coinfected patients undergoing TDF-containing ART. Liver fibrosis progression and regression were evaluated as endpoints with respect to an extensive list of determinants: host characteristics (age, alcohol consumption etc.), HIV and HBV viral suppression, immunosuppression, antiretroviral and antiviral therapy, biomarkers related to liver-related disease and HBV infection and surrogates of metabolic disorders. We also intended to examine the impact of baseline fibrosis on achieving therapeutic endpoints [12], namely undetectable HBV-DNA and seroclearance of hepatitis B “e” antigen (HBeAg) and hepatitis B surface antigen (HBsAg) during therapy.

## Methods

### Patients and study design

Patients from the French HIV–HBV cohort were included in the present study, as described previously [13]. Briefly, a total of 308 patients were recruited from seven centres located in Paris and Lyon, France during May 2002–May 2003. Inclusion criteria were HIV-positive serology confirmed by western blot and HBsAg-positive serology for at least six months. Patients were prospectively followed every six to twelve months until 2010–2011. All patients provided written informed consent to participate and the protocol was approved by the appropriate ethics committee, in accordance with the Helsinki Declaration.

Patients in this sub-study were included provided that they initiated TDF-containing ART during follow-up. Patients were not included if they had any one of the following: positive HCV-RNA by a sensitive polymerase chain reaction (PCR)-based assay, positive HDV serology, did not have at least two study visits while undergoing TDF-containing ART, discontinued TDF six months after initiation and did not have available fibrosis measurements at TDF-initiation and at least once during follow-up.

### HBV virological and serological parameters

Plasma HBV-DNA viral load (VL) was quantified at cohort inclusion and every six to twelve months using a commercial PCR-based assay (COBAS®AmpliPrep/COBAS®TaqMan®, detection limit: 12 IU/mL or COBAS®Amplicor HBV Monitor, detection limit: 60 IU/mL; Roche Diagnostics, Meylan, France). Due to varying detection thresholds, undetectable HBV-DNA was defined at the highest threshold (HBV-VL < 60 IU/mL). HBV mutations at position rt204 were determined using DNA chip technology, as described previously [14].

Qualitative HBsAg, HBeAg and anti-HBe antibodies were detected at cohort inclusion and every yearly visit using a commercial enzyme immunoassay. *HBeAg-seroclearance* was defined as any patient with HBeAg-loss during follow-up and *HBeAg-seroconversion* was defined as HBeAg-loss and acquiring anti-HBe antibodies. *HBsAg-seroclearance* was defined as HBsAg-loss during follow-up.

### HIV-related virological and immunological parameters

Plasma HIV-1 RNA VLs were measured at cohort inclusion and every six months using either a branched-DNA (b-DNA Quantiplex 3.0, detection limit: 50 copies/mL, Bayer Diagnostics, Cergy Pontoise, France) or real-time PCR technique (COBAS AmpliPrep/COBAS TaqMan HIV-1 test, detection limit: 40 copies/mL, Roche Molecular Systems, Meylan, France). CD4+ T-cell counts were quantified at cohort inclusion and every six months using standard measurements, while nadir CD4+ cell count was obtained from patient records prior to inclusion.

### Assessing liver enzymes and fibrosis

Alanine aminotransferase (ALT) and aspartate aminotransferase (AST) levels were quantified using standard methods for every study visit. ALT and AST levels were regrouped in relation to the upper limit normal (ULN), defined at 35 IU/mL: <1× ULN, 1–2× ULN and >2× ULN. Liver fibrosis was assessed at each yearly interval by the FibroTest® calculated from a standard battery of biochemical levels [15]. METAVIR equivalents of these measures, established in the HIV–HBV coinfected population, were used to grade liver fibrosis [16] (F2: 0.48–0.58, F3: 0.59–0.73, F4: ≥0.74).

### Assessing alcohol consumption, cardiovascular disease and diabetes

Patients were asked at cohort inclusion and every twelve months whether they drank alcohol and if so, how many glasses per day, week or month were consumed on average over the past year. Alcohol consumption was then divided into three categories: no consumption, >0–2 glasses/day and >2 glasses/day. Patients were considered to have cardiovascular disease (CVD) if they were treated with an agent indicated for CVD (cardiac therapy, antihypertensives, diuretics, peripheral vasodilators, beta blockers, calcium channel blockers, ACE inhibitors, angiotensin antagonists or lipid-modifying agents) or were diagnosed by their treating physician with any hypertensive, ischaemic or other forms of heart disease. Patients were considered diabetic if they were treated with insulin, insulin analogues or a blood glucose lowering agent or were diagnosed by their treating physician with diabetes.

### Statistical analysis

Baseline was defined as the study visit at which TDF was commenced. Follow-up began at baseline and continued until treatment discontinuation, loss to follow-up, final visit of the cohort study or death, whichever occurred first.

Baseline characteristics were first compared between patients with F0–F1–F2 (none, mild or moderate fibrosis) versus F3–F4 (severe fibrosis/cirrhosis) liver fibrosis using the Kruskal–Wallis test for continuous variables and Pearson’s *χ* ^2^ test or Fisher’s exact test for categorical variables. Differences in time to undetectable HBV-DNA (among patients with detectable HBV-DNA at baseline) and HBeAg-seroclearance (among patients with HBeAg-positive serology at baseline) were also compared between baseline fibrosis groups using Kaplan–Meier curves and were tested using Cox proportional hazards models.

In a longitudinal evaluation of liver fibrosis, we compared patients with baseline F0–F1–F2 levels progressing to F3–F4 at the end follow-up versus no progression, as well as patients with baseline F3–F4 levels regressing to F0–F1–F2 versus no regression. Comparisons were made using the same statistics as mentioned above. Since liver fibrosis levels are known to vary substantially over time, we also examined the determinants of transitioning from F0–F1–F2 to F3–F4 and from F3–F4 to F0–F1–F2 between study visits. Transition rates were estimated from homogenous continuous-time Markov models. Univariable hazards ratios (HR) were calculated for time-fixed and time-varying covariables using maximum likelihood methods. Risk factors with *P *< 0.05 in univariable analysis were used to create a predictive, multivariable model in forward-stepwise fashion.

Statistical analysis was performed using STATA (v12.1, College Station, TX) and R (v3.2.0, Vienna, Austria), while significance was determined using a *P* value < 0.05.

## Results

### Description of the study population at baseline

Of the 308 patients enrolled, 237 had one study visit at which TDF-containing ART was administered. Of them, 70 were excluded due to one of the following reasons: HCV-RNA positive and/or HDV seropositive (*n *= 36), did not have at least two consecutive study visits while undergoing TDF-containing ART (*n *= 12), discontinued TDF-containing ART six months after initiation (*n *= 4), and did not have available fibrosis at baseline (*n *= 12) and at least once during follow-up (*n *= 6). In total, 167 patients were included in analysis.

As shown in Table 1, roughly two-thirds of patients were HBeAg positive and almost 80% had detectable HBV-DNA at baseline with a median level of 4.93 log_10_ IU/mL. Prevalence of other comorbidities, such as excessive alcohol consumption and diabetes, were low with the exception of CVD at 16.2%. Almost all patients had previous exposure to ART at the time of TDF-initiation. TDF was administered in combination with other nucleotide/nucleoside reverse transcriptase inhibitors (*n *= 21), non-nucleotide/nucleoside reverse transcriptase inhibitors (NNRTI, *n *= 53), PIs (*n *= 62), integrase inhibitors (*n *= 1) or both NNRTIs and PIs (*n *= 30). Atazanavir (ATZ) use at TDF initiation or at some point during follow-up was observed in 43 (25.7%) patients.

**Table 1.** Description of the study population at TDF initiation.

		Liver fibrosis levels at TDF initiation		
	Total	F0–F1–F2	F3–F4	
	(*n *= 167)	(*n *= 120)	(*n *= 47)	*P*^c^
**Demographics**				
Sex ratio (males/females) (% males)	143/24 (85.6)	96/24 (80.0)	47/0 (100)	<0.001
Age (years)^b^	42 (36–48)	40 (35–45)	44 (41–53)	<0.001
BMI (kg/m^2^)^b^ [*N* = 161]	22.3 (20.9–24.5)	22.8 (21.0–24.8)	21.5 (20.4–23.1)	0.006
Originating from high HBV-endemic zone^a^	39 (23.4)	36 (30.0)	3 (6.4)	0.001
Alcohol consumption (glasses/day)^b^ [*N* = 151]	0 (0–2)	1 (0–2)	0 (0–2)	0.13
Cardiovascular disease^a^	27 (16.2)	16 (13.3)	11 (23.4)	0.11
Diabetes^a^	4 (2.4)	3 (2.5)	1 (2.1)	0.9
**HIV infection**				
Duration of known HIV infection (years)^b^	11.0 (6.0–14.7)	10.2 (5.3–13.7)	12.7 (8.6–15.8)	0.002
AIDS-defining illness^a^	47 (28.1)	26 (21.7)	21 (44.7)	0.003
CD4+ cell count (/mm^3^)^b^ [*N* = 166]	405 (295–565)	402 (299–557)	475 (253–576)	0.8
CD4+ cell count (/mm^3^)^a^ [*N* = 166]				0.03
≥500	57 (34.3)	39 (32.5)	18 (39.1)	
≥350 and <500	46 (27.7)	40 (33.3)	6 (13.0)	
<350	63 (38.0)	41 (34.2)	22 (47.8)	
Nadir CD4+ cell count (/mm^3^)^b^ [*N* = 154]	217 (102–321)	226 (108–326)	194 (82–307)	0.3
HIV-RNA (<50 copies/mL)^a^ [*N* = 165]	95 (57.6)	64 (53.8)	31 (67.4)	0.11
HIV-RNA (log_10_ copies/mL)^b,e^	3.75 (2.69–4.53)	3.98 (2.78–4.72)	3.74 (2.43–4.29)	0.3
ART-naïve^a^	3 (1.8)	3 (2.5)	0	0.3
Duration of ART (years)^b,d^	6.7 (4.1–9.2)	6.0 (3.8–8.6)	7.8 (6.4–10.6)	0.004
Previous antiretroviral exposure^a,d^				
Zidovudine	137 (83.5)	91 (77.8)	46 (97.9)	0.001
Stavudine	104 (63.4)	68 (58.1)	36 (76.6)	0.03
Didanosine	99 (60.4)	67 (57.3)	32 (68.1)	0.2
Zalcitabine	42 (25.6)	23 (19.7)	19 (40.4)	0.006
Nevirapine	26 (15.9)	19 (16.2)	7 (14.9)	0.8
Efavirenz	74 (45.1)	53 (45.3)	21 (44.7)	0.9
Indinavir/r	75 (45.7)	46 (39.3)	29 (61.7)	0.009
Saquinavir/r	29 (17.7)	19 (16.2)	10 (21.3)	0.4
**HBV characteristics**				
Duration of known HBV infection (years)^b^	8.0 (3.8–12.2)	6.9 (3.5–10.8)	10.9 (6.2–15.0)	0.003
Undetectable HBV-DNA (<60 IU/mL)^a^	32 (19.3)	25 (21.0)	7 (14.9)	0.4
HBV-DNA (log_10_ copies/mL)^b,e^	4.93 (2.94–7.15)	5.33 (2.95–7.24)	4.42 (2.75–6.60)	0.16
HBeAg positive^a^	104 (62.3)	74 (61.7)	30 (63.8)	0.8
Previous LAM-exposure^a,d^	148 (90.2)	103 (88.0)	45 (95.7)	0.16
Cumulative LAM duration (months)^b,f^	56.0 (33.1–76.4)	50.9 (31.0–71.6)	73.0 (51.3–85.2)	<0.001
Concomitant LAM/FTC-treatment^a^	120 (71.9)	84 (70.0)	36 (76.6)	0.4
ALT (IU/mL)^b^ [*N* = 164]	43 (28–72)	40 (24–69)	47 (31–74)	0.3
AST (IU/mL)^b^ [*N* = 164]	36 (27–58)	32 (25–52)	48 (32–73)	0.003
AST/ALT^b^ [*N* = 164]	0.86 (0.66–1.11)	0.84 (0.60–1.10)	0.89 (0.74–1.24)	0.10

^a^Number (%). ^b^Median (IQR). ^c^Significance between fibrosis groups determined using Kruskal–Wallis test for continuous variables and Pearson’s *χ* ^2^ test or Fisher’s exact test for categorical variables.

^d^Among ART-experienced patients. ^e^Among patients with detectable HIV or HBV viremia.

^f^Only among patients with previous LAM exposure.

When comparing patients with F3–F4 (*n *= 47, 28.1%) versus F0–F1–F2 (*n *= 120, 71.9%) liver fibrosis, the former group was more likely to be male, older, not from a region of high HBV-endemicity and have lower body mass index (BMI) (Table 1). Of note, 83.3% of females came from a region of high HBV-endemicity. Patients with F3–F4 fibrosis were more likely to have a CD4+ cell count <350/mm^3^, an AIDS-defining illness, and longer duration of ART and known HIV infection. Previous exposure to zidovudine (AZT), stavudine (D4T), zalcitabine (DDC) and ritonavir-boosted indinavir (IDV/r) was also more frequent in the group with baseline F3–F4 fibrosis, while no significant differences in prior exposure to other potentially hepatotoxic agents was observed [nevirapine (*P *= 0.9), efavirenz (*P *= 0.9), lopinavir/r (*P *= 0.15)]. Finally, significantly longer duration of known HBV-infection, longer cumulative exposure to lamivudine (LAM), and higher AST levels were observed in patients with F3–F4 fibrosis.

### Baseline fibrosis levels and HBV-related endpoints

**Figure 1.**
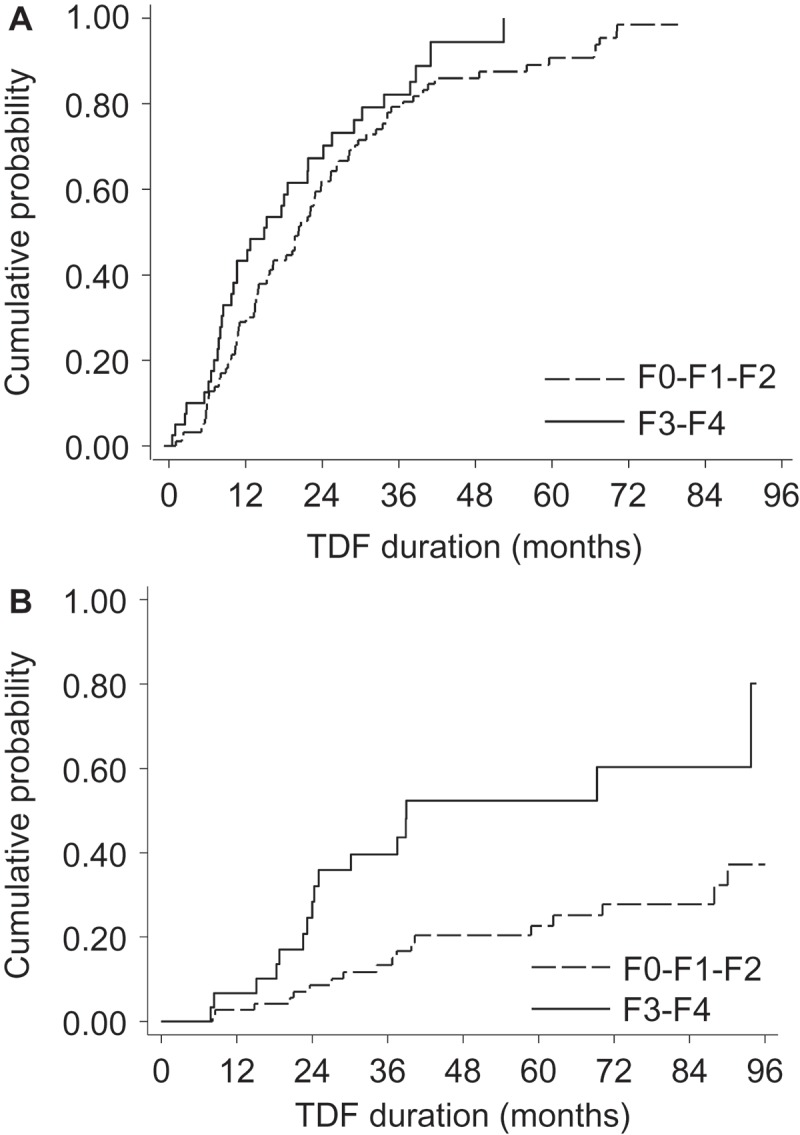
Baseline liver fibrosis levels and HBV-related endpoints during tenofovir-containing antiretroviral therapy. Kaplan–Meier curves are used to depict time to HBV-related endpoints during tenofovir (TDF) containing ART, stratified on baseline none/mild/moderate liver fibrosis (F0–F1–F2) and fibrosis/cirrhosis (F3–F4). Time to undetectable HBV-DNA (<60 IU/mL) is represented in (a) among patients with detectable HBV-DNA at baseline. Time to HBeAg-seroclearance is represented in (b) among patients with HBeAg-positive serology at baseline.

Overall, patients were followed for a median sixty months (IQR = 36–93). Among the 134 patients with detectable HBV-DNA VL at baseline, undetectable HBV-DNA was achieved in 44 (32.8%) at year one, 80 (59.7%) at year two, 101 (75.4%) at year three and 118 (88.1%) at the end of follow-up (median seventeen months until undetectable HBV-DNA, IQR = 10–29). As shown in Figure 1(a), time to achieving undetectable HBV-DNA was shorter in patients with baseline F3–F4 versus F0–F1–F2 fibrosis (median 13 versus twenty months, respectively), yet there was no significant difference (*P *= 0.11) even after adjusting for baseline HBV-DNA VL (*P *= 0.18).

Among the 104 patients with HBeAg-positive serology at baseline, 35 (33.7%) had HBeAg-seroclearance after twenty-nine months (IQR = 21–40), among whom 13 (37.1%) had HBeAg-seroconversion. As shown in Figure 1(b), time to HBeAg-seroclearance was shorter in patients with baseline F3–F4 versus F0–F1–F2 fibrosis (median twenty-four versus thirty-seven months, respectively, *P *<0.001). A significant association was maintained after adjusting for potential factors influencing HBeAg-seroconversion (age, baseline HBV-DNA, baseline elevations in ALT and CD4+ count as a time-dependent covariate, *P *= 0.001).

In total, five (3.0%) patients had HBsAg-seroclearance after a median twenty-three months (range = 9–53) of treatment. The small number of HBsAg-seroclearance events precluded any formal statistical comparison between baseline liver fibrosis groups.

### Evolution of liver fibrosis during tenofovir-containing ART

**Figure 2.**
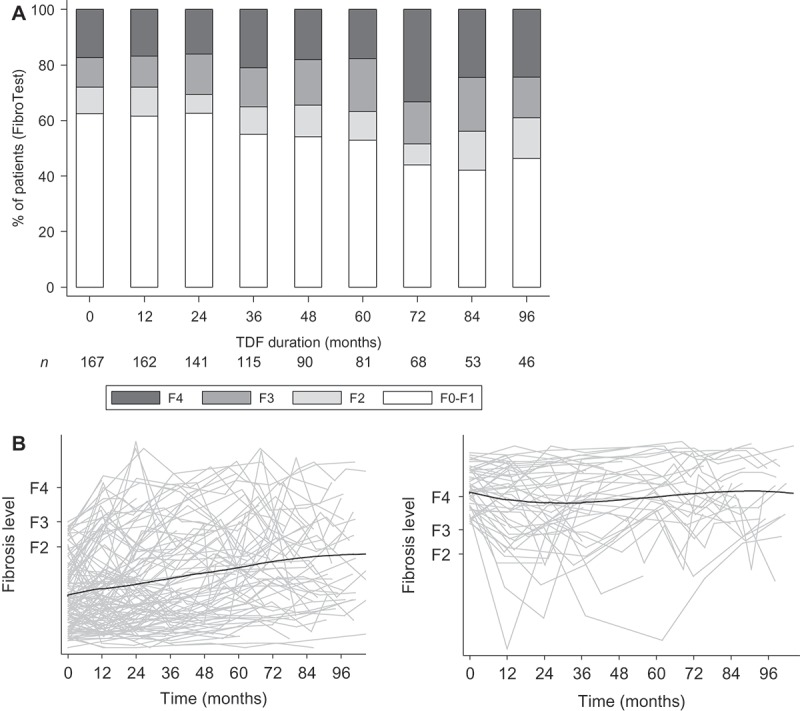
Liver fibrosis evolution during tenofovir-containing antiretroviral therapy. In (a), the distribution of liver fibrosis levels is provided at each yearly interval during tenofovir (TDF) containing ART. The number of patients continuing follow-up at the end of each interval is provided below. In (b), individual trajectories of FibroTest® scores in function of their METAVIR fibrosis equivalents are provided in grey lines for patients with <F3 and ≥F3 baseline fibrosis (left and right panels, respectively), while LOWESS plots are given as black lines to depict moving averages over time.

In total, 939 liver fibrosis assessments with the FibroTest® were conducted during follow-up. Liver fibrosis levels are summarized at each year of TDF-containing ART in Figure 2(a), while average FibroTest® scores are given over time in Figure 2(b).

In patients with F3–F4 baseline liver fibrosis, 7/47 (14.9%) regressed to F0–F1–F2 fibrosis at last follow-up visit. Regression occurred a median nine months (IQR = 8–17) after TDF initiation. Patients with fibrosis regression were significantly more likely to have a higher CD4+ cell count both at treatment initiation and at the end of follow-up (Table 2). At baseline, there were no significant differences between groups with respect to liver-related parameters (i.e. platelet counts, albumin etc.). Most of these parameters remained stable until the end of follow-up, yet marked improvements in hyaluronic acid were observed in both groups and significantly higher prothrombin time at last follow-up visit was found in patients with fibrosis regression (Table 2).

**Table 2.** Description of patients with severe fibrosis/cirrhosis at baseline regressing to F0–F1–F2 fibrosis by the end of follow-up.

	No regression	Regression to F0–F1–F2	
	(*n *= 40)	(*n *= 7)	*P*^c^
**At baseline**			
Male gender^a^	40 (100)	7 (100)	*Ntp*
Age >40 years^a^	34 (85.0)	7 (100)	0.6
BMI (kg/m^2^)^b^	21.5 (20.5–23.2)	20.8 (19.4–22.0)	0.3
Zone of high HBV-endemicity^a^	3 (7.5)	0	0.9
Alcohol consumption (glasses/day)^a^			0.4
0	25 (65.8)	3 (42.9)	
>0–2	7 (18.4)	2 (28.6)	
>2	6 (15.8)	2 (28.6)	
Cardiovascular disease^a^	10 (25.0)	1 (14.3)	0.5
Diabetes^a^	1 (2.5)	0	0.9
Fasting glycaemia (mmol/L)^b^	5.1 (4.6–5.8)	5.1 (4.2–5.4)	0.4
Fasting triglycerides (mmol/L)^b^	1.87 (1.52–2.92)	0.98 (0.59–1.62)	0.007
AIDS-defining illness^a^	19 (47.5)	2 (28.6)	0.4
CD4+ cell count (/mm^3^)^b^	400 (213–565)	576 (540–759)	0.009
CD4+ cell count (≥350/mm^3^)^a^	21 (53.9)	7 (100)	0.03
Nadir CD4+ cell count (/mm^3^)^b^	158 (78–304)	305 (216–346)	0.13
Nadir CD4+ (≥250/mm^3^)^a^	13 (35.1)	4 (66.7)	0.19
HIV-RNA (<50 copies/mL)^a^	26 (66.7)	5 (71.4)	0.9
ART duration (years)^b^	8.0 (6.2–10.6)	7.5 (6.6–9.2)	0.9
PI-containing ART^a^	22 (55.0)	2 (28.6)	0.2
HBV-DNA (log_10_ IU/mL)^b^	3.32 (2.31–5.18)	6.58 (1.78–7.38)	0.7
HBV-DNA <60 IU/mL^a^	5 (12.5)	2 (28.6)	0.3
HBeAg-positive^a^	25 (62.5)	5 (71.4)	0.9
ALT >2 × ULN^a^	8 (21.1)	3 (42.9)	0.3
Prothrombin time^b^	90 (82–96)	96 (84–100)	0.12
Platelet count (10^9^/L)^b^	160 (114–201)	195 (146–245)	0.16
<150 (10^9^/L)^a^	18 (45.0)	2 (28.6)	0.7
Albumin (g/L)^b^	44 (39–46)	45 (40–47)	0.5
<36 g/L^a^	3 (7.7)	0	0.9
Hyaluronic acid (µg/mL)^b^	58 (30–119)	49 (33–81)	0.7
Previous LAM use^a^	38 (95.0)	7 (100)	0.9
Mutations at position rt204^a,^^d^	19 (47.5)	4 (57.1)	0.6
**At end of follow-up**			
TDF duration (months)^b^	72 (37–94)	47 (23–94)	0.3
HBV-DNA (<60 IU/mL)^a^	32 (80.0)	6 (85.7)	0.9
HBeAg-seroclearance^a,^^e^	10 (40.0)	2 (40.0)	0.9
HBsAg-seroclearance^a^	2 (5.0)	0	0.9
ALT >2 × ULN^a^	3 (7.5)	0	0.9
AST >2 × ULN^a^	2 (5.0)	0	0.9
Prothrombin time^b^	90 (83–98)	97 (95–100)	0.03
			
Platelet count (10^9^/L)^b^	167 (115–213)	201 (147–238)	0.3
<150 (10^9^/L)^a^	16 (40.0)	2 (28.6)	0.7
Albumin (g/L)^b^	43.1 (40.7–47.0)	46.0 (40.8–49.0)	0.4
<36 g/L^a^	2 (5.1)	0	0.9
Hyaluronic acid (µg/mL)^b^	43 (23–67)	33 (10–57)	0.4
Fasting glycaemia (mmol/L)^b^	5.1 (4.9–5.4)	5.0 (4.9–5.1)	0.5
Fasting triglycerides (mmol/L)^b^	1.64 (1.21–3.00)	1.07 (0.85–2.05)	0.09
HIV-RNA (<50 copies/mL)^a^	36 (90.0)	7 (100)	0.9
Change in CD4+ cell count^b^	18 (−63, 184)	10 (−84, 39)	0.7
CD4+ cell count (≥500/mm^3^)^a^	13 (33.3)	6 (85.7)	0.01

^a^Number (%). ^b^Median (IQR).

^c^Significance between regression groups determined using Kruskal–Wallis test for continuous variables and Pearson’s *χ* ^2^ test or Fisher’s exact test for categorical variables. *ntp*: no test performed.

^d^Patients without documented resistance were considered not to have any rt204 mutations.

^e^Only HBeAg-positive patients; four patients without regression seroreverted HBeAg-positive after seroclearance.

In patients with F0–F1–F2 baseline fibrosis, 20/120 (16.7%) progressed to F3–F4 fibrosis at last follow-up visit. Progression occurred a median twenty-six months (IQR = 19–52) after TDF initiation. Patients with fibrosis progression were significantly more likely to be male, older than forty years of age, born in a low/moderate HBV-endemic country and have lower nadir CD4+ cell count (Table 3). Patients with liver fibrosis progression were also more likely to be anaemic at TDF initiation. At the end of follow-up, levels of liver-related biochemical parameters improved in most patients and no significant differences were observed between those with or without fibrosis progression (Table 3).

**Table 3.** Description of patients without fibrosis/cirrhosis at baseline progressing to F3–F4 fibrosis by the end of follow-up.

	No progression (*n *= 100)	Progression to F3–F4 (*n *= 20)	*P*^c^
**At baseline**			
Male gender^a^	76 (76.0)	20 (100)	0.01
Age >40 years^a^	43 (43.0)	17 (85.0)	0.001
BMI (kg/m^2^)^b^	22.7 (21.0–24.8)	23.6 (21.4–24.5)	0.4
Zone of high HBV-endemicity^a^	35 (35.0)	1 (5.0)	0.007
Alcohol consumption (glasses/day)^a^			0.10
0	44 (50.6)	5 (26.3)	
>0–2	22 (25.3)	9 (47.4)	
>2	21 (24.1)	5 (26.3)	
Cardiovascular disease^a^	12 (12.0)	4 (20.0)	0.5
Diabetes^a^	3 (3.0)	0	0.9
Fasting glycaemia (mmol/L)^b^	4.9 (4.6–5.4)	5.2 (5.0–5.6)	0.03
Fasting triglycerides (mmol/L)^b^	1.30 (0.90–2.09)	1.46 (1.06–1.89)	0.9
AIDS-defining illness^a^	20 (20.0)	6 (30.0)	0.3
CD4+ cell count (/mm^3^)^b^	404 (320–576)	367 (229–520)	0.19
CD4+ cell count (≥350/mm^3^)^a^	71 (71.0)	10 (50.0)	0.07
Nadir CD4+ cell count (/mm^3^)^b^	237 (116–365)	186 (32–242)	0.03
Nadir CD4+ (≥250/mm^3^)^a^	43 (45.7)	2 (11.8)	0.01
HIV-RNA (<50 copies/mL)^a^	52 (52.5)	12 (60.0)	0.6
ART duration (years)^b^	5.9 (3.4–8.6)	6.8 (5.5–9.9)	0.2
PI-containing ART^a^	40 (40.0)	12 (60.0)	0.10
HBV-DNA (log_10_ IU/mL)^b^	4.55 (2.17–6.88)	2.95 (2.42–5.27)	0.4
HBV-DNA (<60 IU/mL)^a^	22 (22.2)	3 (15.0)	0.6
HBeAg-positive^a^	60 (60.0)	14 (70.0)	0.5
ALT >2 × ULN^a^	23 (23.2)	4 (20.0)	0.9
Prothrombin time^b^	93 (86–100)	90 (83–100)	0.6
Platelet count (10^9^/L)^b^	202 (170–247)	163 (135–243)	0.11
<150 (10^9^/L)^a^	13 (13.1)	8 (40.0)	0.004
Albumin (g/L)^b^	43.0 (39.7–46.0)	43.5 (40.0–45.5)	0.6
<36 g/L^a^	13 (13.0)	0 (0)	0.12
Hyaluronic acid (µg/mL)^b^	31 (19–53)	35 (22–70)	0.2
Previous LAM-use^a^	85 (85.0)	19 (95.0)	0.3
Mutations at position rt204^a,^^d^	41 (41.0)	6 (30.0)	0.4
**At end of follow-up**			
TDF duration (months)^b^	48 (28–86)	63 (26–100)	0.18
HBV-DNA (<60 IU/mL)^a^	79 (79.0)	18 (90.0)	0.4
HBeAg-seroclearance^a,^^e^	11 (18.3)	6 (42.9)	0.05
HBsAg-seroclearance^a,^^f^	2 (2.0)	0	0.9
ALT >2 × ULN^a^	4 (4.0)	3 (15.0)	0.06
AST >2 × ULN^a^	3 (3.1)	2 (10.0)	0.2
Prothrombin time^b^	95 (91–100)	90 (84–100)	0.2
Platelet count (10^9^/L)^b^	207 (182–246)	179 (161–229)	0.18
<150 (10^9^/L)^a^	12 (12.1)	3 (15.0)	0.7
Albumin (g/L)^b^	43.0 (39.7–46.2)	41.2 (39.8–43.7)	0.10
<36 g/L^a^	6 (6.3)	0	0.6
Hyaluronic acid (µg/mL)^b^	24 (16–32)	31 (16–47)	0.16
Fasting glycaemia (mmol/L)^b^	5.0 (4.7–5.4)	5.6 (5.0–5.8)	0.008
Fasting triglycerides (mmol/L)^b^	1.15 (0.93–1.67)	1.38 (0.97–2.49)	0.16
HIV-RNA (<50 copies/mL)^a^	83 (83.8)	19 (95.0)	0.3
Change in CD4+ cell count^b^	139 (−14, 243)	66 (−53, 220)	0.5
CD4+ cell count (≥500/mm^3^)^a^	59 (60.2)	8 (40.0)	0.10

^a^Number (%). ^b^Median (IQR).

^c^Significance between progression groups determined using Kruskal–Wallis test for continuous variables and Pearson’s *χ* ^2^ test or Fisher’s exact test for categorical variables.

^d^Patients without documented resistance were considered not to have any rt204 mutations.

^e^Only HBeAg-positive patients; two patients without progression seroreverted HBeAg-positive after seroclearance.

^f^One patient without progression seroreverted HBsAg-positive after seroclearance.

Transient episodes of liver fibrosis regression were observed in 13/47 (27.7%) patients with baseline F3–F4 fibrosis, while transient episodes of fibrosis progression were observed in 17/120 (14.2%) patients with baseline F0–F1–F2 fibrosis. When examining changes in liver fibrosis status between follow-up visits (over a possible 730 transitions), the majority of transitions indicated no change in liver fibrosis levels (82.6%). In multivariable analysis, transitions involving liver fibrosis progression were significantly associated with higher age, male gender, longer ART-duration and concomitant PI-containing ART (Table 4). Of note, AIDS-defining illness was a significant determinant in univariable analysis, yet was no longer significant after adjustment (adjusted-HR = 1.59, 95% CI = 0.90–2.81). No risk factors associated with transitions involving liver fibrosis regression was identified (Table 4).

**Table 4.** Determinants of transitioning to and from none/mild/moderate liver fibrosis (F0–F1–F2) and severe fibrosis/cirrhosis (F3–F4) during tenofovir-containing ART.

	Univariable		Multivariable^a^	
Determinant	F0–F1–F2 → F3–F4	F3–F4 → F0–F1–F2	F0–F1–F2 → F3–F4	F3–F4 → F0–F1–F2
**Age at baseline**				
per year	1.08 (1.05–1.12)	0.96 (0.92–1.00)		
>40 years	3.75 (2.09–6.72)	0.69 (0.36–1.29)	1.08 (1.04–1.12)	0.96 (0.92–1.01)
Female gender	0.18 (0.05–0.61)	1.94 (0.58–6.51)	0.16 (0.05–0.57)	1.43 (0.36–5.71)
Zone of high HBV-endemicity	0.24 (0.11–0.53)	0.80 (0.34–1.90)		
Cardiovascular disease	1.88 (1.12–3.18)	0.81 (0.46–1.44)		
Diabetes	5.30 (1.25–22.41)	1.28 (0.29–5.72)		
AIDS-defining illness	2.29 (1.36–3.85)	0.86 (0.49–1.51)		
CD4+ cell count				
≥500/mm^3^ at baseline	0.90 (0.54–1.48)	0.98 (0.58–1.68)		
≥350/mm^3^ at baseline	0.64 (0.39–1.05)	1.13 (0.65–1.95)		
≥500/mm^3^ during follow-up	0.69 (0.42–1.15)	1.11 (0.65–1.89)		
≥350/mm^3^ during follow-up	0.79 (0.46–1.35)	1.19 (0.65–2.16)		
Nadir CD4+ cell count (≥250/mm^3^)	0.63 (0.36–1.08)	1.01 (0.57–1.78)		
HIV-RNA (<50 copies/mL)	1.49 (0.79–2.81)	1.12 (0.53–2.35)		
Previous antiretroviral exposure				
Zidovudine	2.38 (1.07–5.30)	0.72 (0.25–2.04)		
Stavudine	1.40 (0.84–2.34)	0.61 (0.35–1.06)		
Indinavir/r	1.73 (1.06–2.81)	1.17 (0.68–2.01)		
ART duration (per year)	1.12 (1.05–1.19)	1.01 (0.94–1.08)	1.07 (1.00–1.15)	1.01 (0.93–1.09)
PI-containing ART	2.43 (1.44–4.09)	1.22 (0.69–2.17)	2.41 (1.38–4.19)	1.21 (0.64–2.28)
ATZ exposure	3.59 (1.86–6.94)	1.25 (0.63–2.47)		
HBV-DNA viral load				
per log_10_ IU/mL during follow-up	0.93 (0.80–1.08)	1.04 (0.89–1.22)		
<60 IU/mL during follow-up	1.14 (0.68–1.92)	0.93 (0.51–1.68)		
HBeAg positive at baseline	1.34 (0.79–2.29)	1.43 (0.79–2.59)		
ALT >2 × ULN	1.92 (0.96–3.85)	1.74 (0.79–3.84)		

^a^In the multivariable model, continuous age was preferred over age greater than forty years and PIs as a class was preferred over individual agents. In order to avoid overfitting, diabetes was not included. The following variables were removed from the model because their corresponding *P* value was no longer significant (*P *<0.05): zone of high HBV-endemicity, cardiovascular disease, AIDS-defining illness and previous exposure to zidovudine or ritonavir-boosted indinavir.

### Tenofovir discontinuation and HBV-related parameters

During follow-up, 15 patients discontinued TDF after a median twenty-eight months (IQR = 11–34). Reasons for treatment discontinuation were as follows: renal-associated toxicity (*n *= 6), switched treatment due to HIV-resistance (*n *= 2), poor adherence (*n *= 1), lipid abnormality (*n *= 1), pregnancy (*n *= 1), nevirapine-associated Lyell’s syndrome (*n *= 1), possible drug–drug interaction with another antiretroviral agent (*n *= 1), patient’s decision (*n *= 1) and treatment simplification (*n *= 1). Eight patients (53.3%) were able to reinitiate TDF a median 0.5 years (IQR = 0.3–1.7) after discontinuation.

Of those who discontinued, 11 (73.3%) remained treated with an anti-HBV agent (LAM + adefovir, *n *= 2; LAM, *n *= 9) and four (26.7%) did not. A >1.0 log_10_ IU/mL increase in HBV-DNA replication occurred in five (33.3%) patients after TDF discontinuation (four of whom switched to anti-HBV-containing ART), while two (13.3%) patients were unable to achieve controlled HBV-DNA replication during TDF and after TDF-discontinuation (both switched to ART not containing an anti-HBV agent). Only three (20.0%) had a >2× increase in ALT levels from their previous visit. Two of nine patients with baseline F0–F1–F2 fibrosis levels increased to F3–F4 fibrosis after TDF discontinuation, while one of six patients with baseline F3–F4 regressed to F0–F1–F2 and later re-progressed to F3–F4 liver fibrosis upon discontinuation.

### Liver-related morbidity and mortality during tenofovir

At baseline, three patients (1.8%) had a liver-related event prior to TDF initiation: portal hypertension (*n *= 2) and hepatic failure (*n *= 1). These patients remained alive throughout their follow-up (range = nineteen to forty-seven months). During follow-up, four patients had a liver-related event (IR = 0.5/100 person-years) after a range of nine to sixty-nine months. These events included: portal hypertension (*n *= 2), HCC (*n *= 1) and unspecified liver disease (*n *= 1). Overall, there were three deaths during follow-up: one patient with HCC died of a myocardial infarction, one was the result of severe pneumonia and the last one was due to an AIDS-related illness.

## Discussion

In this prospective study, we observed that the majority of HIV–HBV coinfected patients remained at the same level of fibrosis when undergoing tenofovir-containing ART for up to nine years. In patients with severe fibrosis and cirrhosis, regression to mild or moderate liver fibrosis was observed in 15% and generally occurred during the first years of TDF. This result mirrors other short-term findings from TDF-treated coinfected patients with high levels of baseline fibrosis [8,9]. With longer follow-up, any improvement in fibrosis appears minimal. We also observed, surprisingly, that 17% of patients with low-level liver fibrosis at baseline progressed to F3–F4 fibrosis, particularly at later years of follow-up. Since a noninvasive score was used to stage fibrosis, progression/regression could be partly attributed to changes in circulating markers linked more closely to necroinflammation and not necessarily fibrosis [17].

Liver fibrosis is a major driving factor for severe clinical outcomes such as HCC, which, if it occurs, leads to rapid progression to death [18]. Higher levels of liver stiffness measures and biochemical scores are predictive of these events during treatment in HBV mono-infected patients [19,20], even for those with subclinical cirrhosis [21] or sustained virological response [22]. Despite our observations that almost a fifth of patients exhibited increases of liver fibrosis during tenofovir, the values of these noninvasive scores would indicate low-risk of any liver-related event. Accordingly, we found a rare incidence of HCC and death associated with liver disease during TDF-treatment, which was much lower compared to other cohorts of coinfected patients with suboptimal antiviral treatment [23], suggesting some clinical benefit with this treatment strategy. Studies with larger numbers of patients would still be of great benefit to determine the exact relationship of TDF, HBV-replication and liver-related mortality in the context of HIV–HBV.

Higher fibrosis levels at baseline did not appear to influence virological response in our cohort; however, patients with advanced fibrosis did have significantly faster rates of HBeAg-seroclearance. Similar findings have been observed in HBV mono-infected patients treated with potent anti-HBV agents [24,25]. HBeAg-seroclearance is known to be driven by higher levels of inflammation during the natural course of HBV infection [26]. Although there was no significant difference in ALT levels between baseline fibrosis groups, γ-glutamyl transferase, hyaluronic acid, AST and platelet counts were significantly higher in those with advanced baseline fibrosis, which could account for the more accelerated HBeAg-seroclearance rate observed in our study.

In HBV mono infection, it is well established that liver fibrosis decreases in the vast majority of patients treated with highly potent nucleos(t)ide analogues (NAs), whether evaluated by liver biopsies [4,27,28] or transient elastography (TE) [29,30]. As the patients in our study were all coinfected, the degree of HIV-induced immunosuppression could play a substantial role in profibrogenic processes and hepatocyte regeneration [11]. Indeed, we observed that having higher baseline CD4+ cell counts were indicative of fibrosis regression by the end of follow-up. Previous AIDS-defining illness was associated with transitions to fibrosis progression in univariable analysis and lower nadir CD4 cell count in patients with baseline F0–F1–F2 fibrosis were associated with progression to F3–F4 fibrosis by the end of follow-up. The immunological components giving rise to fibrosis have been explored in a previous study among coinfected patients with severe immunosuppression. After initiating ART containing an anti-HBV agent, these patients exhibited negligible changes in intrahepatic T cell and natural killer cell activation and consistently increased levels of intrahepatic apoptosis, all of which are implicated in liver fibrosis [31]. Taken together, these findings highlight the need to initiate ART early-on during infection and maintain adequate levels of CD4+ T cells.

The finding on fibrosis progression is rather concerning since it is fairly rare in HBV mono-infected TDF-treated patients [4]. Coinfected patients with liver fibrosis progression had significantly higher levels of fasting glycaemia at the end of follow-up and those with regression had significantly lower levels of triglycerides at TDF-initiation – the median levels of both parameters were nonetheless borderline normal or slightly abnormal in these patient groups. Furthermore, patients progressing to severe fibrosis/cirrhosis had a higher, albeit non-significant, proportion with elevated liver enzymes at the end of follow-up, which has been associated with NASH, insulin resistance and liver fibrosis in HIV-mono-infected patients [32]. These results point to preliminary development of metabolic abnormalities as a possible underlying cause for some of the liver fibrosis progression observed here, yet would require further evaluation in other studies.

Furthermore, one noticeable risk factor associated with transitions to severe fibrosis/cirrhosis in patients with F0–F1–F2 liver fibrosis at baseline was exposure to protease inhibitors. When looking at individual agents, none of the PI combinations classically associated with hepatotoxicity during coinfection with chronic viral hepatitis, such as ritonavir-boosted saquinavir or IDV/r [33], were linked to liver fibrosis progression, but instead ATZ. This agent does inhibit uridine diphosphate-glucuronil transferase, frequently causing drug-induced increases of bilirubin [34]. Since the FibroTest® uses bilirubin as part of its score, a slight overestimation of fibrosis levels likely resulted among patients undergoing ATZ, potentially causing a spurious association between ATZ and liver fibrosis. To mitigate any confounding from this PI, we did adjust time trends of liver fibrosis during treatment with ATZ use (Supporting Figure 1) and still observed generally stable levels of liver fibrosis over time. Nevertheless, some residual measurement bias could remain.

Other antiretroviral agents appeared to influence liver fibrosis levels particularly at baseline. Patients with F3–F4 fibrosis at treatment initiation were more likely to have previous exposure with IDV, D4T and AZT. All of these agents are known to modify the risk of abdominal lipohypertrophy, hepatic steatosis/nonalcoholic steatohepatitis (NASH) and/or insulin resistance [35], which again implicates metabolic disorders in fibrosis progression.

The host factors age and male gender have been traditionally strong determinants of liver fibrosis progression for a wide range of liver diseases [36]. Likewise, in our study, increased age was associated with liver fibrosis progression and males were more likely to transition to F3–F4 fibrosis. The differences in gender follow closely with previous research, in which estradiol, associated with reduced apoptosis of hepatocytes, activation of hepatic stellate cells and suppressed hepatic fibrosis, are produced at higher levels in premenopausal females and could provide protection against fibrosis development [37]. Alternatively, most females originated from sub-Saharan countries of high HBV prevalence and thus could have been at stages of less-active infection with lower risk of liver fibrosis progression [38]. Other host factors, such as BMI and alcohol consumption, could not be properly evaluated in this study due to the restricted and rather “healthy” distribution of these variables (3% BMI >30 kg/m^2^ and 3% >5 glasses/day of alcohol consumption).

Several limitations of our study need to be addressed. First, liver fibrosis was determined by a noninvasive marker, which contains a certain degree of measurement error and uncertainly in its ability to predict fibrosis progression and/or regression. Combining TE and biochemical scores has been shown to improve predictive capacity of liver fibrosis [39]. We did have TE measurements available in the source cohort, yet they were collected more frequently at later visits and were unable to be consistently used in this study. Second, we did not collect specific data on steatosis, insulin levels or NASH and hence are limited in fully evaluating their implication in liver fibrosis. Third, there could be additional measurement error in other variables. Assessment of alcohol consumption was limited to the average number of drinks during an extended period of time, and might not have accounted for past or irregular patterns of drinking. Some of the agents used to define CVD have multiple indications and by including them, could have overestimated CVD in this cohort. Fourth, differential bias in loss to follow-up could have explained some observations; however, baseline characteristics were similar between patients completing follow-up versus lost to follow-up (Supporting Table 1) or between patients with more than versus less than eight years of TDF-containing ART (Supporting Table 2).

Lastly, our data represent a population that, prior to initiating TDF, had more extensive ART experience and more severe immunosuppression compared to contemporary patient populations. However, as the clinical profiles of patients in this study are still actively seen in out-patient settings, these data highlight a target group likely requiring more extensive care, such as the use of liver biopsies for identifying other important pathologies (i.e. NASH). No data on liver fibrosis evolution exist to date in treatment-naïve patients initiating TDF or tenofovir alafenamide and hence validation of these findings would be warranted.

## Conclusions

Liver fibrosis, as determined by a validated noninvasive surrogate, decreases in a small minority of HIV–HBV coinfected patients during TDF. These observations are for the most part unrelated to HBV, considering the extensive control of HBV-replication. Since past levels of immunosuppression are strongly associated with liver fibrosis progression, earlier ART initiation would be a priority during HIV–HBV coinfection. Meanwhile, the effect of metabolic disorders on liver fibrosis, to the extent that our study could demonstrate, should be elucidated in further research. Finally, our data stress the importance of continuous liver fibrosis monitoring as part of routine care in this patient group.

## Supplementary material

To access the supplementary material to this article please see Supplementary Files under Article Tools online.
